# Supplementary material for: Self-assembling nanofibrous bacteriophage microgels as sprayable antimicrobials targeting multidrug-resistant bacteria
Source: Nat Commun. 2022 Dec 5;13:7158. doi: 10.1038/s41467-022-34803-7 (PMC9723106; doi:10.1038/s41467-022-34803-7)
Supplement: Supplementary file 1 — Supplementary information [file 41467_2022_34803_MOESM1_ESM.pdf]

Supplementary Information:

Self-Assembling Nanofibrous Viral Microgels as Sprayable Antimicrobials Targeting Multidrug-Resistant Bacteria

Lei Tian,[1] Leon He, [1] Kyle Jackson,[1] Ahmed Saif, [1] Shadman Khan, [2] Zeqi Wan,[1] Tohid F. Didar [2,3,4], Zeinab Hosseinidoust [1,2,3]*

[1] Department of Chemical Engineering, McMaster University, Hamilton, Ontario, L8S 4L7, Canada

[2] School of Biomedical Engineering, McMaster University, Hamilton, Ontario, L8S 4K1, Canada

[3] Michael DeGroote Institute for Infectious Disease Research, McMaster University, Hamilton, Ontario, L8S 4K1, Canada

[4] Department of Mechanical Engineering, McMaster University, Hamilton, Ontario, L8S 4L7, Canada

[*]Corresponding Author. Phone: (905) 525-9140; email: doust@mcmaster.ca

Supplementary Note 1. The number of reactive functional groups on the M13 phage capsid

The number of reactive subset of amine and carboxylic groups from on the M13 coat proteins were calculated based on the amino acid sequence summarized from reference[1]. M13 capsid is composed of approximately 2700 copies of pVIII protein, and there are reactive amine groups from 2 lysine and 1 alanine (N-terminal) on each pVIII protein[1]. Therefore, there should be approximately 8,100 reactive amine groups (2,700×3).

The reactive carboxyl groups are provided from 2 aspartic acids and 2 glutamic acids in each pVIII protein[1]. Therefore, there are approximately 10,800 reactive carboxyl groups (2,700×4).

Supplementary Note 2. Evaluating preparation efficiency of phage microgels

Firstly, the size of honeycomb film was measured before microgel isolation (Supplementary Fig. 3a). After collecting all the microgels into 1 mL of water from the peeled template, we dropped 5 μL of microgel suspension on the glass slide and snapped high-resolution images (Supplementary Fig. 3b-c). The amount of microgels in that droplet was then counted, and the microgel preparation efficiency was calculated based on the equation (1):

$$\eta = N_{microgel} \times 200/S_{template} \qquad (1)$$

where η is the microgel preparation efficiency, $N_{microgel}$ is the amount of microgels in that droplet, and $S_{template}$ is the size of honeycomb film.

Supplementary Note 3. Test of specific targeting ability and desiccation sensitivity of phages

For example, phages M13 (*Inoviridae*, filamentous), HER262 (*Myoviridae*, long tailed), and T7 (*Podoviridae*, short tailed), are all *E. coli* phages and they cannot infect other bacterial species such as *Staphylococcus aureus* (Supplementary Fig. 5). From the three *E. coli* strains we evaluated, M13 phage formed mild lysis zones on the lawns of *E. coli* ER2738 and *E. coli* BL21, while phage HER262 showed lysis zones on strains ER2738 and O157:H7. Phage T7 was able to significantly lyse *E. coli* ER2738 and *E. coli* BL21. It is noteworthy that the highly specific bactericidal action of phages means that to control the population of multiple species, a mixture of phages has to be used[2]. However, the advantage of this specific killing action is preserving the beneficial bacteria in food that are responsible for maintaining the taste and texture of many food products[3,4].

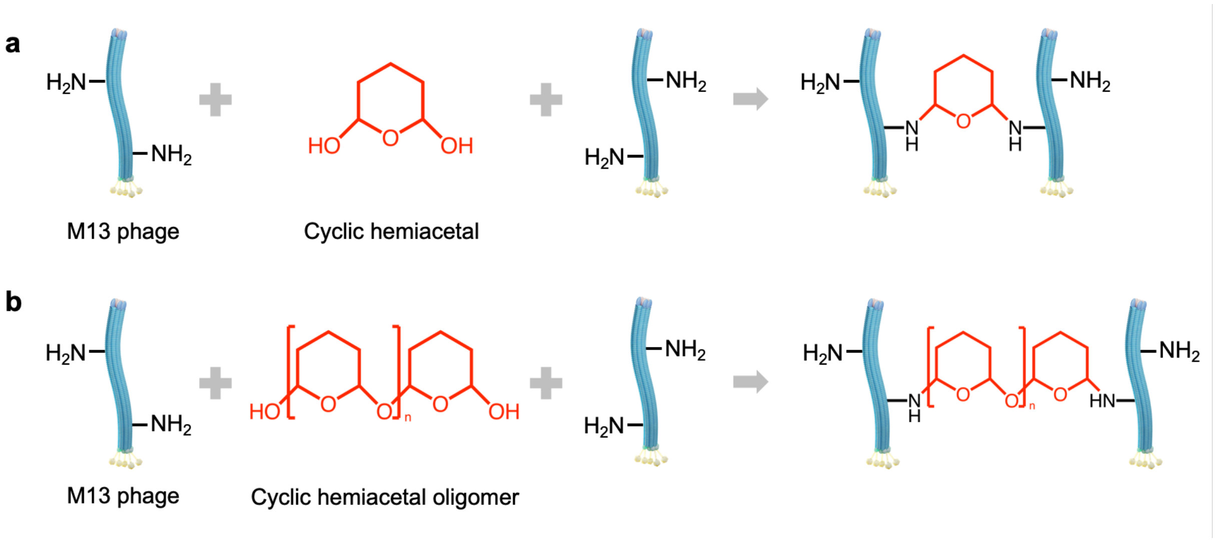

Supplementary Figure 1. The crosslinking reactions between M13 phage and the other formations of GA in aqueous solution. a, Two M13 phages react with cyclic hemiacetal and incorporate. **b**, Two M13 phages react with cyclic hemiacetal and incorporate.

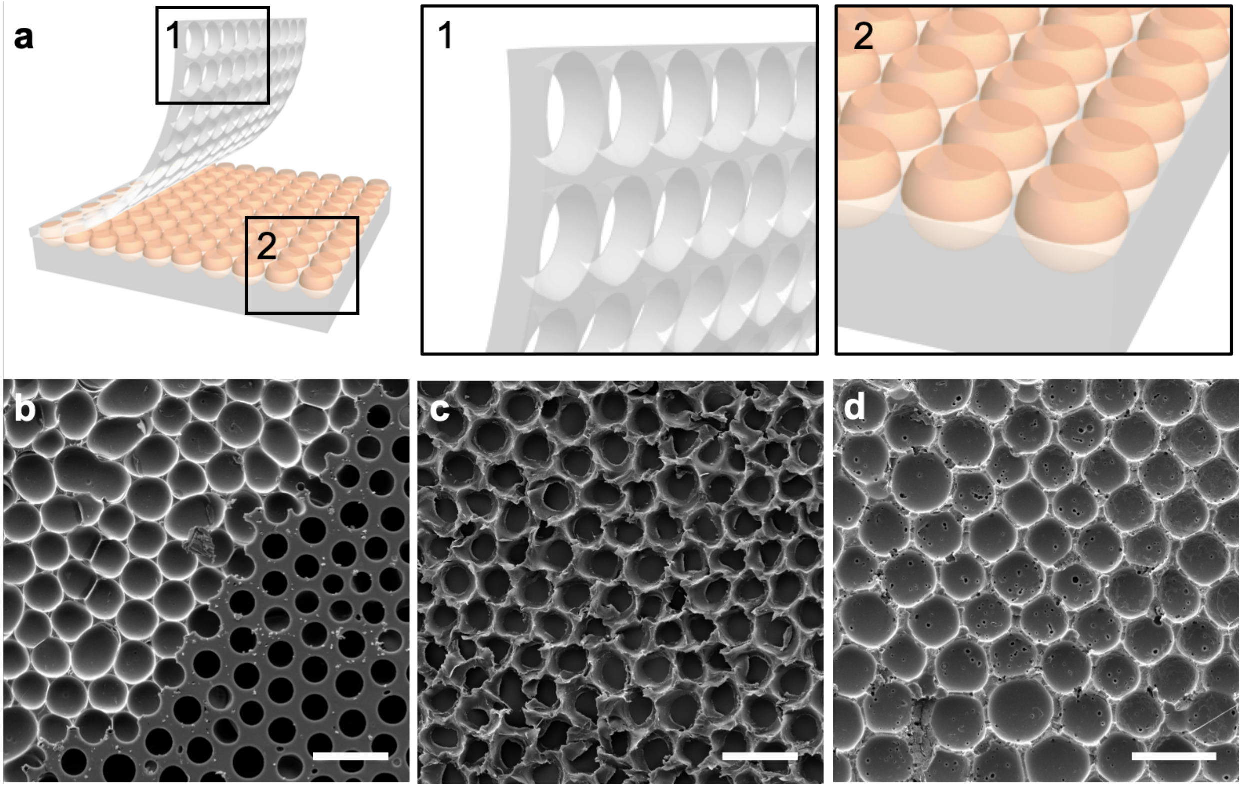

Supplementary Figure 2. The isolation of phage microgels. a, Detailed Schematic image of the peeling process: the top half of the pores was removed and the microgels inside the film were exposed on the bottom film layer without damage. **b**, SEM image of the edge of peeling area of the honeycomb film: The peeled area is on the top left presenting shallow pores and the unpeeled area is on the bottom right showing deep spherical pores. **c**, Large-scale SEM image of the adhesive tape after peeling. Scale bar: 50 μm. **d**, Large-scale SEM image of the honeycomb film after peeling; Scale bar: 50 μm. (n=3 independent experiments in **b-d**)

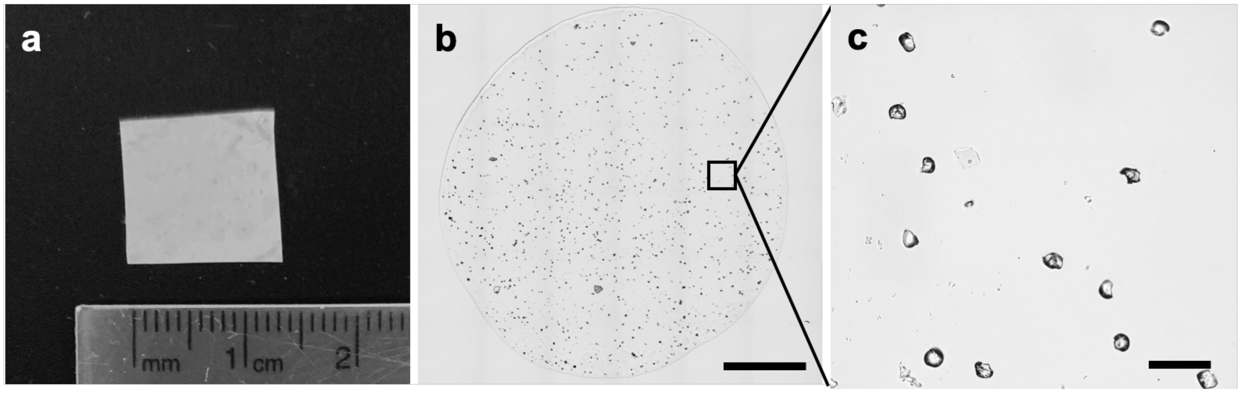

Supplementary Figure 3. The evaluation of microgel preparation efficiency. a, Image of the honeycomb film with microgels inside with rule for film area measurement. **b**, Bright field image of 5 μL of phage microgel suspension for microgel count. Scale bar: 1 mm. **c**, Zoom-in bright field image of **b** showing free-stand microgels. Scale bar: 100 μm. (n=3 independent experiments in **b-c**)

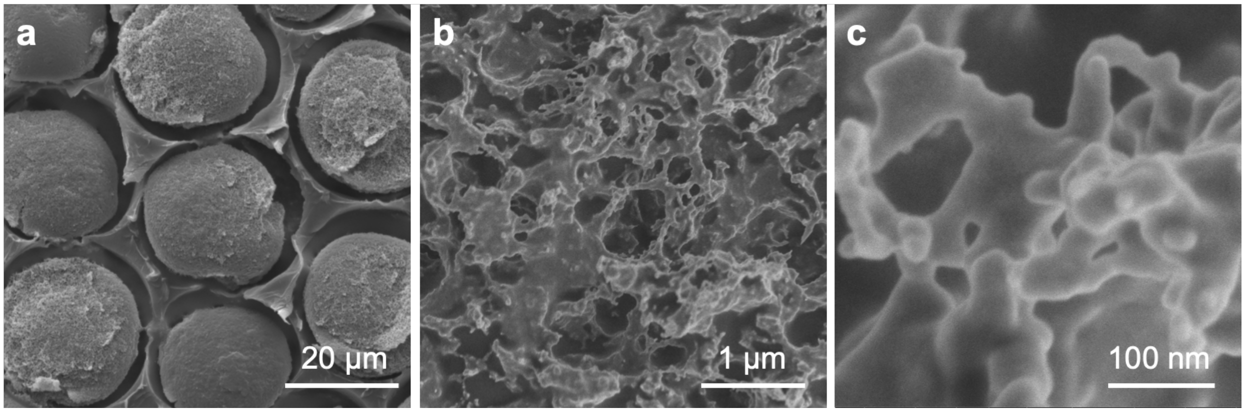

Supplementary Figure 4. Nanostructure of the BSA microgel. a, SEM image of BSA microgels crosslinked by GA in peeled honeycomb template. **b,** Nanostructure on the surface of a BSA microgel. **c,** Zoom-in image of image **b**. (n=3 independent experiments)

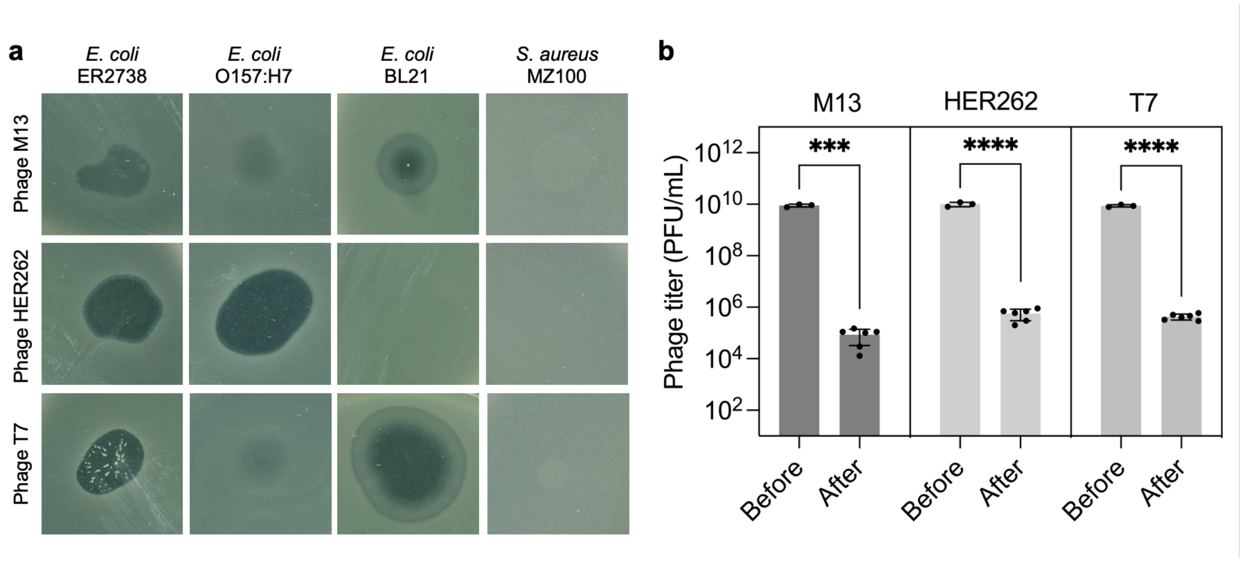

Supplementary Figure 5. Specific infectivity and desiccative sensitivity of phage M13, HER262 and T7. a, Infectivity of phage M13, HER262 and T7 to four types of bacterial strains. **b,** Titer count of phage M13, HER262 and T7 before (n=3 independent experiments for each type of phage) and after desiccation (n=6 independent experiments for each type of phage) for 1 h (M13: p=0.0005; HER262 and T7: p<0.0001). Date are presented as mean±SD with all data points. Statistical significance in all panels is derived from unpaired t-test. ***p<0.001, ****p<0.0001.

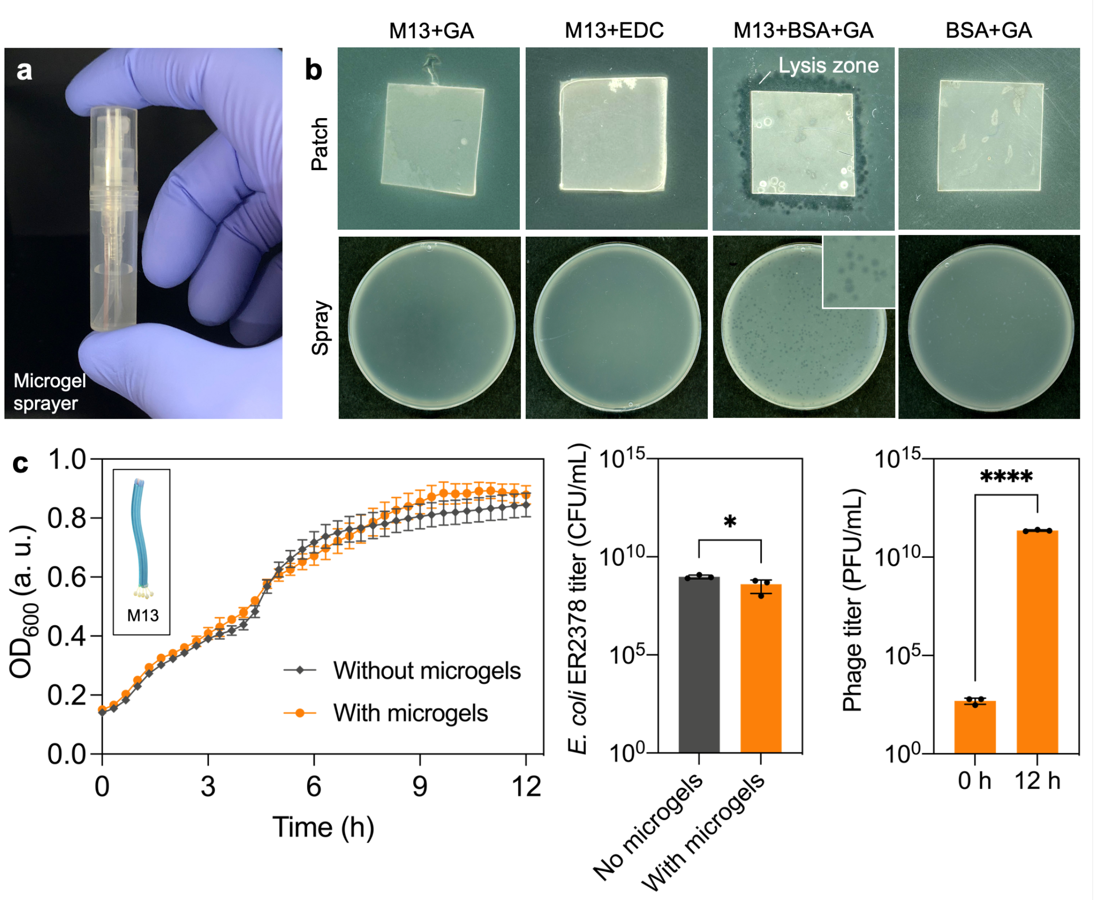

Supplementary Figure 6. Antimicrobial property of different M13 phage microgels. a, Photo of the sprayer containing phage microgel suspension. **b,** Patches and sprayed microgels on the lawn of *E. coli* ER2738. GA: glutaraldehyde; EDC: 1-Ethyl-3-(3-dimethylaminopropyl) carbodiimide; BSA: bovine serum albumin. **c,** Left: Growth curve of *E. coli* ER2738 in LB solution with and without adding microgels. Box on the top left: Schematic of the phage component in these microgels. Middle: Titer count of *E. coli* ER2738 after incubating 12 hours in LB with and without microgels (p=0.0434); Right: Titer count of phage M13 in the *E. coli* ER2738 LB solution with microgels at 0 and 12 h (p<0.0001). Date are presented as mean±SD. n=3 independent experiments. Statistical significance in all panels is derived from unpaired t-test. *p<0.05, ****p<0.0001.

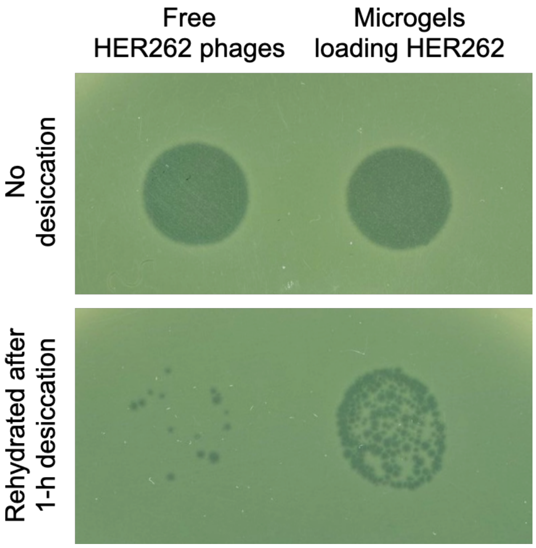

Supplementary Figure 7. Bioactivity of 1$\times$10^6 PFU mL^{-1} of free HER262 phages and M13+HER262+BSA+GA hybrid phage microgels (before and after air-drying for 1 hr) on *E. coli* O157:H7 lawn.

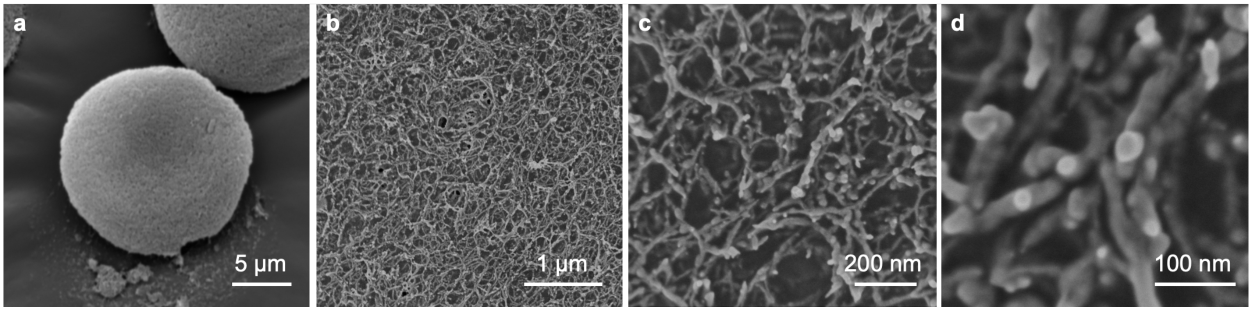

Supplementary Figure 8. Nanostructure of M13+HER262+BSA+GA hybrid phage microgels. a, SEM images of a M13+HER262+BSA+GA microgel. **b-d,** SEM images of nanostructure on the surface of the microgel. (n=3 independent experiments)

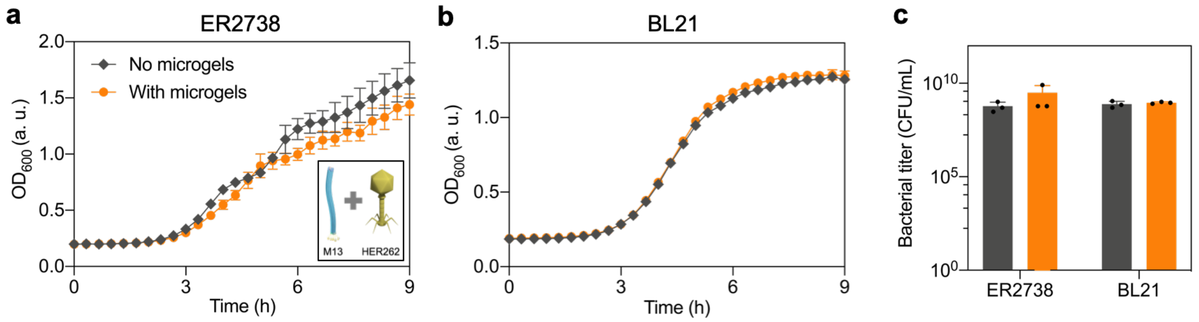

Supplementary Figure 9. M13+HER262+BSA+GA hybrid phage microgels incubating with other *E. coli* strains. a, Optical density (OD) growth of *E. coli* ER2738 (10^6 CFU mL^{-1}) incubated in TSB during 9 hours with and without phage HER262 microgels. Box on the bottom right: Schematic of the 2 phage components in these microgels. a.u.: arbitrary unit. **b,** Optical density growth of *E. coli* BL21 (10^6 CFU mL^{-1}) incubated in TSB during 9 hours with and without phage HER262 microgels. **c,** Final titer count of the *E. coli* ER2738 and BL21 incubated in TSB after 9 hours in figure a-b. (Date are presented as mean±SD, n=3 independent experiments)

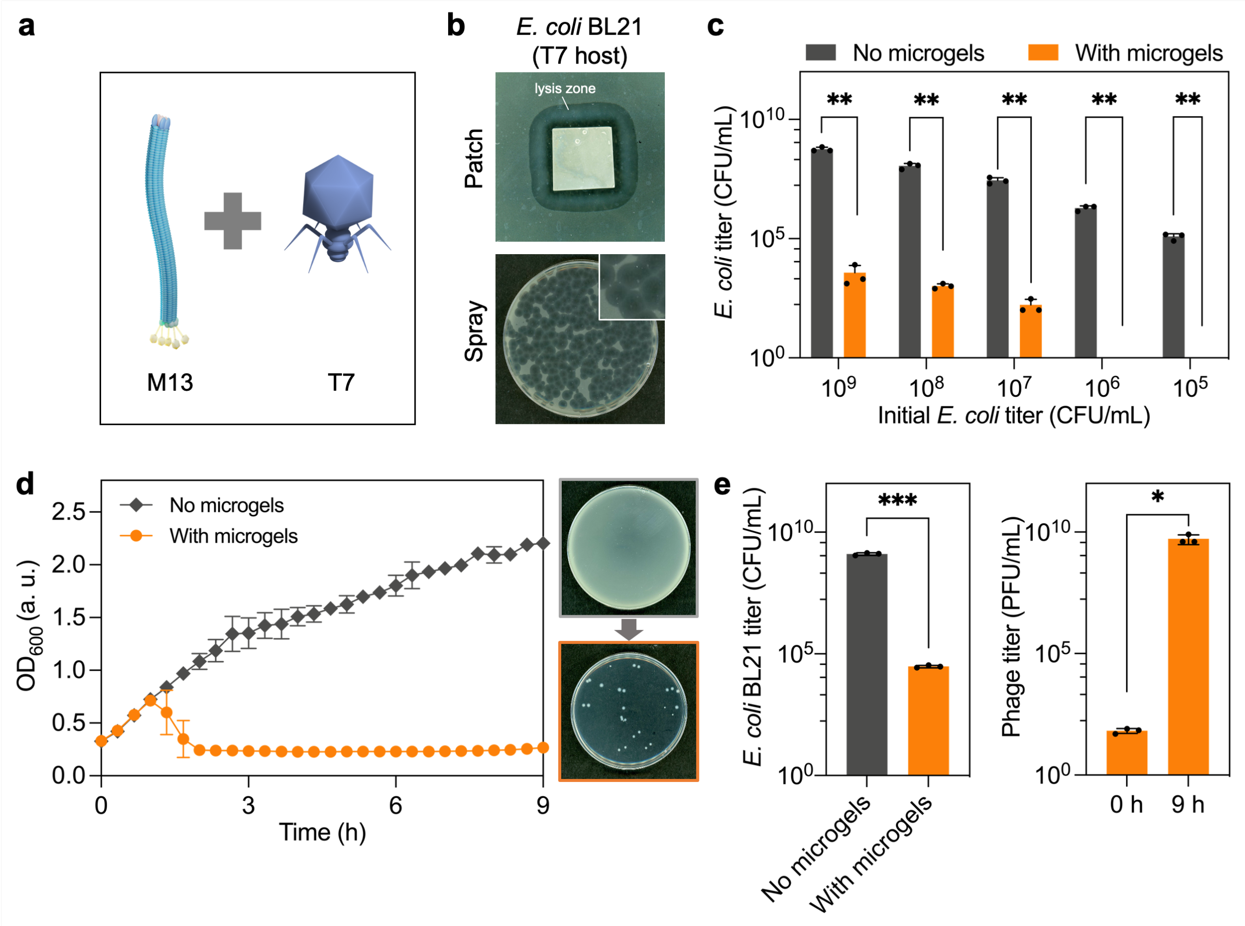

Supplementary Figure 10. Antimicrobial property of M13+T7+BSA+GA hybrid phage microgels. a, Schematic of the 2 phage components in these microgels. **b,** Photos of the hybrid T7 microgels in the patches and spray on the lawn of *E. coli* BL21. **c,** Titer count of *E. coli* BL21 incubated in PBS after 9 hours at different initial concentration with and without T7 microgels. (n=3 independent experiments for each initial concentration. p=0.0011, 0.0030, 0.0051, 0.0019, and 0.0049 respectively) **d,** Left: Optical density growth of *E. coli* ER2738 (10^6 CFU mL^{-1}) incubated in TSB during 9 hours with and without T7 microgels. Right: Compact bacterial lawn and sporadic colonies formed by the bacterial solution incubated without and with microgels respectively. **e,** Left: Titer count of BL21 in TSB after 9 hours incubated with and without microgels (p=0.0001). Right: Titer count of T7 in TSB after 9 hours incubated with microgels (p=0.0161). (Date are presented as mean ± SD. n=3 independent experiments. Statistical significance in all panels is derived from unpaired t-test.*P<0.05, **P<0.01, ***P<0.001)

Supplementary References:

1. Chung, W. J., Lee, D. Y. & Yoo, S. Y. Chemical modulation of M13 bacteriophage and its functional opportunities for nanomedicine. *Int. J. Nanomedicine* **9**, 5825–5836 (2014).
2. Kifelew, L. G. *et al.* Efficacy of phage cocktail AB-SA01 therapy in diabetic mouse wound infections caused by multidrug-resistant Staphylococcus aureus. *BMC Microbiol.* **20**, 1–10 (2020).
3. Guenther, S. & Loessner, M. J. Bacteriophage biocontrol of Listeria monocytogenes on soft ripened white mold and red-smear cheeses. *Bacteriophage* **1**, 94–100 (2011).
4. Perera, M. N., Abuladze, T., Li, M., Woolston, J. & Sulakvelidze, A. Bacteriophage cocktail significantly reduces or eliminates Listeria monocytogenes contamination on lettuce, apples, cheese, smoked salmon and frozen foods. *Food Microbiol.* **52**, 42–48 (2015).
