# Supplementary material for: Self-assembling nanofibrous bacteriophage microgels as sprayable antimicrobials targeting multidrug-resistant bacteria
Source: Nat Commun. 2022 Dec 5;13:7158. doi: 10.1038/s41467-022-34803-7 (PMC9723106; doi:10.1038/s41467-022-34803-7)
Supplement: Supplementary file 3 — Reporting Summary [file 41467_2022_34803_MOESM3_ESM.pdf]

nature portfolio

Corresponding author(s): Zeinab Hosseinidoust

Last updated by author(s): Nov 5, 2022

Reporting Summary

Nature Portfolio wishes to improve the reproducibility of the work that we publish. This form provides structure for consistency and transparency in reporting. For further information on Nature Portfolio policies, see our Editorial Policies and the Editorial Policy Checklist.

Statistics

For all statistical analyses, confirm that the following items are present in the figure legend, table legend, main text, or Methods section.

n/a	Confirmed	
☐	☒	The exact sample size (*n*) for each experimental group/condition, given as a discrete number and unit of measurement
☐	☒	A statement on whether measurements were taken from distinct samples or whether the same sample was measured repeatedly
☐	☒	The statistical test(s) used AND whether they are one- or two-sided<br>*Only common tests should be described solely by name; describe more complex techniques in the Methods section.*
☐	☒	A description of all covariates tested
☒	☐	A description of any assumptions or corrections, such as tests of normality and adjustment for multiple comparisons
☐	☒	A full description of the statistical parameters including central tendency (e.g. means) or other basic estimates (e.g. regression coefficient) AND variation (e.g. standard deviation) or associated estimates of uncertainty (e.g. confidence intervals)
☐	☒	For null hypothesis testing, the test statistic (e.g. F, t, r) with confidence intervals, effect sizes, degrees of freedom and P value noted<br>*Give P values as exact values whenever suitable.*
☒	☐	For Bayesian analysis, information on the choice of priors and Markov chain Monte Carlo settings
☒	☐	For hierarchical and complex designs, identification of the appropriate level for tests and full reporting of outcomes
☐	☒	Estimates of effect sizes (e.g. Cohen's d, Pearson's r), indicating how they were calculated

Our web collection on statistics for biologists contains articles on many of the points above.

Software and code

Policy information about availability of computer code

Data collection	The data was collected from Inverted microscopy (NIS-Elements AR 5.30.01), Scanning electron microscopy (TESCAN VEGA-II LSU, FEI Magellan 400), FT-IR Spectrometer (Nicolet 6700). No external custom code or algorithms was used.
Data analysis	The graphing and stat's software we use was Prism 9. No external custom code or algorithms was used.

For manuscripts utilizing custom algorithms or software that are central to the research but not yet described in published literature, software must be made available to editors and reviewers. We strongly encourage code deposition in a community repository (e.g. GitHub). See the Nature Portfolio guidelines for submitting code & software for further information.

Data

Policy information about availability of data

All manuscripts must include a data availability statement. This statement should provide the following information, where applicable:

- Accession codes, unique identifiers, or web links for publicly available datasets
- A description of any restrictions on data availability
- For clinical datasets or third party data, please ensure that the statement adheres to our policy

We have attached a Source Data file (a single Excel file with data for each figure in a separate sheet).

Field-specific reporting

Please select the one below that is the best fit for your research. If you are not sure, read the appropriate sections before making your selection.

☒ Life sciences ☐ Behavioural & social sciences ☐ Ecological, evolutionary & environmental sciences

For a reference copy of the document with all sections, see nature.com/documents/nr-reporting-summary-flat.pdf

Life sciences study design

All studies must disclose on these points even when the disclosure is negative.

Sample size	1. Figure 1d-i: SEM images of the templates and microgels (n=3 independent experiments)<br>2. Figure 1j: Size distribution of the template pores (n=84 pores measured over 3 independent templates) and the phage microgels prepared with GA (n=58 and 57 microgels at hydrated and dried status respectively, measured over 3 independent experiments), EDC (n=45 and 53 microgels at hydrated and dried status respectively, measured over 3 independent experiments) and BSA (n=56 and 54 microgels at hydrated and dried status respectively, measured over 3 independent experiments).<br>3. Figure 1k: Pore density of the template (n=7 independent films) and the produced microgel count from every square centimeter of the template (n=6 independent experiments for each type of microgels).<br>4. Figure 2a, c-e: n=5 independent experiments;<br>5. Figure 3d: M13+GA microgels: n=4 microgels per fluorescent channel. M13+EDC and M13+BSA+GA microgels: n=5 microgels per fluorescent channel.<br>6. Figure 3f-k: n=3 independent experiments;<br>7. Figure 4d: Titer count of E. coli O157:H7 with (n=5 independent experiments per bacterial concentration) and without (n=3 independent experiments per bacterial concentration) HER262 microgels.<br>8. Figure 4e: Kill curves for E. coli O157:H7 suspension (n=3 independent experiments per bacterial concentration);<br>9. Figure 4f: Final titer count with (n=5 independent experiments per bacterial concentration) and without (n=3 independent experiments per bacterial concentration) HER262 microgels.<br>10. Figure 4h: bacterial titer count of the collected bacterial suspension from contaminated lettuces (n=12 independent lettuces per group).<br>11. Figure 4h-i: n=12 independent food product per group;<br>12. Supplementary Figure 2, 3, 4, 8: Microscopy images (n=3 independent experiments);<br>13. Supplementary Figure 5b: Titer count of phage M13, HER262 and T7 before (n=3 independent experiments for each type of phage) and after desiccation (n=6 independent experiments for each type of phage)<br>14. Supplementary Figure 6c, 9, 10: n=3 independent experiments.
Data exclusions	No data was excluded.
Replication	1. Microgel preparation efficiency and diameter measurement were based on 3 independent experiments;<br>2. Food test was based on 2 independent experiments;
Randomization	1. Microgel preparation efficiency and diameter measurement were based on 9 pictures from 3 independent experiments. These images were captured on the sample locations which were randomly selected by 3 co-authors respectively without discussion (Lei Tian, Ahmed Saif and Zeqi Wan).<br>2. Food test: The randomization is not relevant here because we used lettuce/meat chunks cut from the same meat/lettuce product.
Blinding	1. The microgel preparation preparation was performed 3 times. he first two rounds were completed by first author Lei Tian, and the third round was completed by the co-author Leon He without being informed the material type in advance.<br>2. The microgel characterization (SEM) was done by the technician without being informed the microgel type in advance.<br>3. All the antimicrobial tests of microgel were performed twice. The first round was completed by first author Lei Tian, and the second round was completed by the co-author Ahmed Saif without being informed the microgel type in advance.<br>4. The microgel numbers were counted and analyzed by co-author Zeqi Wan without being informed the microgel type in advance.

Reporting for specific materials, systems and methods

We require information from authors about some types of materials, experimental systems and methods used in many studies. Here, indicate whether each material, system or method listed is relevant to your study. If you are not sure if a list item applies to your research, read the appropriate section before selecting a response.

Materials & experimental systems

n/a	Involved in the study
☒	☐ Antibodies
☒	☐ Eukaryotic cell lines
☒	☐ Palaeontology and archaeology
☒	☐ Animals and other organisms
☒	☐ Human research participants
☒	☐ Clinical data
☒	☐ Dual use research of concern

Methods

n/a	Involved in the study
☒	☐ ChIP-seq
☒	☐ Flow cytometry
☒	☐ MRI-based neuroimaging
